# Supplementary figures and images for: Characterization of Collagen Structure in Normal, Wooden Breast and Spaghetti Meat Chicken Fillets by FTIR Microspectroscopy and Histology
Source: Foods. 2021 Mar 6;10(3):548. doi: 10.3390/foods10030548 (PMC7998852; doi:10.3390/foods10030548)

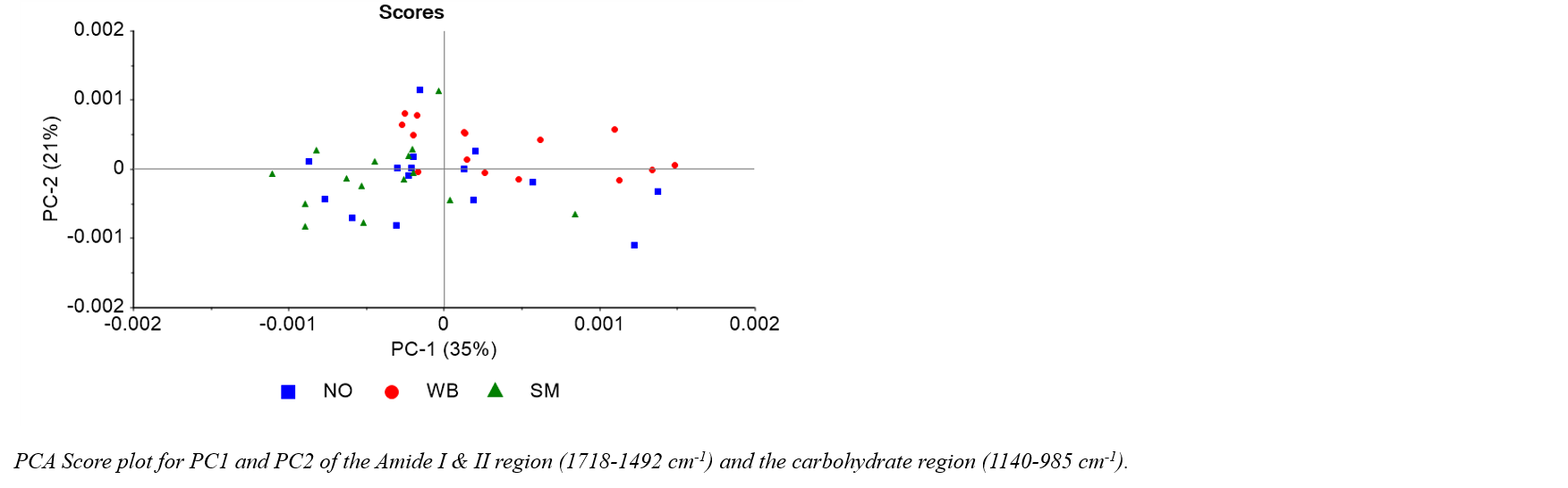

Supplement: Supplementary file 1 [file foods-10-00548-s001.zip › foods-1102603-supplementary.tif]
